# Schwann Cell TRPA1, a Proalgesic Ion Channel, Mediates Neuroinflammation and Fibromyalgia‐Associated Behaviours in Mice

**DOI:** 10.1111/jnc.70424

**Published:** 2026-03-29

**Authors:** Evelyne Silva Brum, Maria Fernanda Pessano Fialho, Daniel Souza Monteiro de Araújo, Lorenzo Landini, Matilde Marini, Francesco De Logu, Romina Nassini, Sara Marchesan Oliveira

**Affiliations:** ^1^ Graduate Program in Biological Sciences: Biochemistry, Department of Biochemistry, Institute of Basic Health Sciences Universidade Federal do Rio Grande do Sul Porto Alegre Rio Grande do Sul Brazil; ^2^ Department of Molecular Pathobiology, Pain Research Center New York University New York New York USA; ^3^ Graduate Program in Neurosciences, Neurobiology Department, Biology Institute Fluminense Federal University Niteroi Rio de Janeiro Brazil; ^4^ Department of Health Sciences, Clinical Pharmacology Unit University of Florence Florence Italy; ^5^ Graduate Program in Biological Sciences: Toxicological Biochemistry, Centre of Natural and Exact Sciences Federal University of Santa Maria Santa Maria Rio Grande do Sul Brazil

## Abstract

Transient receptor potential ankyrin 1 (TRPA1) is an ion channel that integrates the somatosensory system and is specialised in detecting thermal, mechanical, and chemical stimuli. It acts as both a sensor and an amplifier of reactive oxygen and nitrogen species, carbonylic species and lipid peroxidation products, which are overproduced in several painful conditions, including fibromyalgia. Studies have linked TRPA1 to heightened sensitivity to mechanical and cold pain in fibromyalgia patients. In a preclinical mouse model of fibromyalgia induced by reserpine administration, activated Schwann cells expressing TRPA1 trigger an intracellular pathway that leads to the production of reactive oxygen species (ROS) via NADPH oxidase (NOX) 1 and to the recruitment of macrophages in the mouse sciatic and trigeminal nerves. Such mechanisms contribute to mechanical and cold hypersensitivity and early anxiety‐ and depression‐like behaviours. Future translational studies will be essential to validate whether pharmacological modulation of the Schwann cell TRPA1/NOX1 pathway could provide clinical benefit in fibromyalgia.

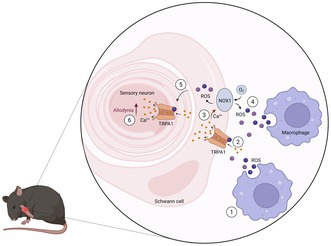

## Graphical abstract

Cellular and molecular mechanisms contributing to TRPA1‐mediated mechanical and cold hypersensitivity and peripheral neuroinflammation in the reserpine‐induced experimental fibromyalgia model. (1) Macrophage‐originated reactive oxygen species (ROS; purple dots) target the transient receptor potential ankyrin 1 (TRPA1) channel localised in Schwann cells. (2) TRPA1 activation in Schwann cells amplifies (3) the calcium (Ca^2+^)‐dependent (orange dots), NADPH oxidase 1 (NOX1)‐mediated ROS (purple dots) production with a dual function. (4) The outward ROS release maintains the macrophage recruitment to the nerve trunk, whereas (5) the inward ROS release targets sensory neuron TRPA1 (6) to produce nociception. Graphical abstract was created in BioRender. Fialho, M. (2025) https://BioRender.com/k66uaca.

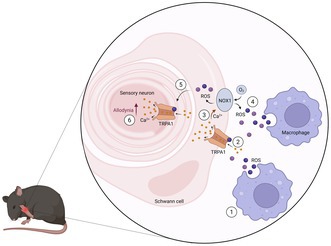

**Abbreviations**
AAPT: ACTTION‐American Pain Society Pain Taxonomy
ACR: American College of Rheumatology
ACTTION: Analgesic, Anesthetic and Addiction Clinical Trial Translations Innovations Opportunities and Networks
ANKTM1: ankyrin‐like protein with transmembrane domains 1
CGRP: calcitonin gene‐related peptide
CNS: central nervous system
DRG: dorsal root ganglia
EULAR: European League Against Rheumatism
FDA: Food and Drug Administration
GABA: γ‐aminobutyric acid
MAFIA: macrophage Fas‐induced apoptosis
NOX: NADPH oxidase
PBN: phenyl‐α‐tert‐butyl nitrone
PNS: peripheral nervous system
RNS: reactive nitrogen species
ROS: reactive oxygen species
TRP: transient receptor potential
TRPA1: transient receptor potential ankyrin 1
TRPM3: transient receptor potential melastatin 3
TRPV1: transient receptor potential vanilloid 1

## 1 TRPA1 Channel, a Sensor of Noxious Stimuli and Oxidative Stress

Transient receptor potential ankyrin 1 (TRPA1) is a member of the transient receptor potential (TRP) channel family, which in mammals includes approximately 30 proteins divided into six subfamilies (Andrade et al. 2012). It was initially identified in 1999 in human foetal lung fibroblasts as an ankyrin‐like protein with transmembrane domains 1 (ANKTM1) (Jaquemar et al. 1999) and was recognised as a TRP channel member only in 2003 due to its homology with other family members (Story et al. 2003). TRPA1 possesses 14–18 ankyrin repeats in the amino‐terminal region, a number much higher than that of other TRPs (0–8 ankyrin repeats), a distinctive feature that inspired its name (Andrade et al. 2012). The complete structure of human TRPA1 was fully elucidated by single‐particle cryo‐electron microscopy in 2015 (Paulsen et al. 2015), which enabled the investigation of TRPA1 regulatory mechanisms and the design of new analgesics and anti‐inflammatory drugs based on its structure.

TRPA1 substantially contributes to nociception perception, as it is mainly expressed in a subpopulation of primary sensory neurons (C‐ and Aδ‐fibres) (Story et al. 2003). More recently, a critical contribution of TRPA1 expressed in non‐neuronal cells has been recognised. In the peripheral nervous system, Schwann cells that ensheath nociceptors express TRPA1 and participate in pain mechanisms (De Logu et al. 2017; De Logu, De Prá, et al. 2020; De Logu et al. 2022). Similarly, in the central nervous system (CNS), TRPA1 is expressed in astrocytes (Takizawa et al. 2018) and oligodendrocytes (Hamilton et al. 2016), where it promotes the maintenance of a pro‐oxidant environment, thereby sustaining inflammation. Beyond the nervous system, TRPA1 has also been identified in a variety of non‐neuronal cells, including epidermal keratinocytes, dermal fibroblasts and melanocytes (Atoyan et al. 2009), synoviocytes (Kochukov et al. 2006) and mast cells (Oh et al. 2013), where it contributes to different regulatory and pro‐inflammatory pathways. These findings support that TRPA1 functions as an important integrator of neuro‐glial and immune signalling, extending beyond its expression in nociceptive neurons.

TRPA1 integrates the somatosensory system, making it specialised for detecting chemical, thermal and mechanical stimuli (Souza Monteiro de Araujo et al. 2020). It is sensitive to the redox state of the milieu (Nakao et al. 2024; Mori et al. 2016), acting as a sensor (Andersson et al. 2008; Trevisani et al. 2007) and amplifier (De Logu et al. 2017) of oxidative stress. This condition is associated with the overproduction of reactive oxygen and nitrogen species (ROS and RNS), carbonylic species and lipid peroxidation products (Forman and Zhang 2021). Originally, TRPA1 was proposed as a cold sensor, specifically within the noxious range (< 17°C) (Story et al. 2003). Indeed, several in vivo studies have established that mice with a genetic deletion of TRPA1 exhibit marked deficiencies in nocifensive responses to noxious cold (Vriens and Voets 2019). However, more recently, it was demonstrated that, together with TRPV1 (vanilloid) and TRPM3 (melastatin), TRPA1 also plays a crucial role in the acute detection of noxious heat (Vandewauw et al. 2018). It seems that, similar to cold responses, TRPA1 heat responses are highly dependent on the cellular environment and are strongly enhanced in a redox state (Moparthi et al. 2016) and by compounds released during tissue injury (Vriens and Voets 2019). Beyond thermal sensing, interest in TRPA1 as a mechanosensor was inspired by the large ankyrin repeat domain in its amino‐terminal region, which potentially acts as an opening‐and‐closing spring of the channel (Andrade et al. 2012). Its role in noxious mechanotransduction, including the development of mechanical allodynia and hyperalgesia, has been elucidated in preclinical models of inflammatory, neuropathic and nociplastic pain (Souza Monteiro de Araujo et al. 2020; Andrade et al. 2012). Taken together, these findings support the concept that TRPA1 acts as a polymodal sensor, with a highly context‐dependent contribution to pain signalling across sensory modalities.

TRPA1 is a nonselective ion channel with high permeability to divalent cations. When TRPA1 is activated, calcium‐dependent pathways are stimulated (Rajagopal and Ponnusamy 2017). Free calcium in the cytosol plays a critical role in several cellular processes, including the release of vasoactive peptides such as substance P and calcitonin gene‐related peptide (CGRP) from peripheral nociceptors (Andrade et al. 2012; Souza Monteiro de Araujo et al. 2020). These peptides are responsible for neurogenic inflammation, characterised by increased vascular permeability, including CGRP‐mediated arteriolar dilation and cellular infiltration. Additionally, CGRP release has been considered a crucial factor in the development of migraine pain (Wattiez et al. 2020). Thus, TRPA1 acts as a key mediator of oedema and of thermal, mechanical and chemical hypersensitivity observed in several painful conditions (Andrade et al. 2012; Landini et al. 2022), highlighting its central role in inflammatory processes and pain.

In this context, using genetic or pharmacological approaches in preclinical models, it has been demonstrated that TRPA1 participates in acute inflammatory painful processes resulting from thermal wounds (de David Antoniazzi et al. 2018) and surgical incisions (Sugiyama et al. 2017), as well as in chronic inflammatory conditions such as complete Freund's adjuvant‐induced arthritis (da Costa et al. 2010) and monosodium iodoacetate‐induced osteoarthritis (Moilanen et al. 2015). Moreover, TRPA1 plays an important function in musculoskeletal pain, including that caused by traumatic muscle injury (Kudsi et al. 2023) and by aromatase inhibitors, such as anastrozole, letrozole and exemestane, adjuvant endocrine treatments for hormone receptor‐positive breast cancer (Fusi et al. 2014; Fialho et al. 2023; De Logu et al. 2016).

Furthermore, the role of TRPA1 has already been identified in neuropathic pain, such as in partial sciatic nerve ligation‐induced nerve injury (De Logu et al. 2017), in streptozotocin‐induced diabetic neuropathic pain (Koivisto and Pertovaara 2013), alcoholic neuropathy (De Logu et al. 2019; Landini, Souza Monteiro de Araujo, et al. 2023), multiple sclerosis caused in relapsing–remitting experimental autoimmune encephalomyelitis (Dalenogare et al. 2020; Rodrigues et al. 2025), complex regional pain syndrome type I evoked by ischemia and reperfusion (De Logu, De Prá, et al. 2020) and in neuropathic pain induced by chemotherapeutic agents such as dacarbazine, paclitaxel, oxaliplatin, cisplatin, bortezomib and thalidomide (Brusco et al. 2019; Materazzi et al. 2012; Nassini et al. 2011; Trevisan et al. 2013; De Logu, Trevisan, et al. 2020; Becker et al. 2023). TRPA1 is substantial for mediating cancer pain as well, including the one caused by thyroid, melanoma, lung and breast tumours growth in the primary site or metastasis (Maqboul and Elsadek 2017; Antoniazzi et al. 2019; De Logu et al. 2021; Landini, Marini, et al. 2023). Additional studies suggest a role for TRPA1 in painful conditions of a nociplastic nature, such as endometriosis (an inflammatory chronic condition associated with nociceptive, neuropathic and nociplastic pain) (Titiz et al. 2024), migraine (De Logu et al. 2022) and fibromyalgia (Achenbach et al. 2019; Brum et al. 2024b, 2025). This evidence highlights the central role of TRPA1 in multiple pain modalities, suggesting that modulation of this channel could have broad therapeutic implications.

## 2 Fibromyalgia: Mechanisms Underlying Symptomatology

Fibromyalgia is a complex clinical disorder with an unknown aetiology, underdiagnosed and undertreated. In 1990, the American College of Rheumatology (ACR) defined criteria for an individual to be diagnosed with fibromyalgia, in which they should present with widespread pain (above and below the head, and on both sides of the body) for at least 3 months, as well as tenderness in more than 11 or 18 tender points (Wolfe et al. 1990). To make it more accurate, several modifications to the ACR's diagnostic criteria have been made, aiming to eliminate the tender point count and to include patient self‐report of pain locations and cognitive difficulties (Wolfe et al. 2010, 2011, 2016). The latest diagnostic criterion was established by the ACTTION‐American Pain Society Pain Taxonomy (AAPT) in 2019 (Arnold et al. 2019). Nowadays, fibromyalgia is the third most common musculoskeletal condition, affecting 2%–3% of the world's population (Sarzi‐Puttini et al. 2020). It can develop at any age, including childhood, but the peak is between 50 and 60 years old (Clauw 2014). At first, women were diagnosed 8–30 times more frequently than men. However, with the advent of new diagnostic criteria that also assess the main comorbidities, a female‐to‐male ratio of 1:1 to 4:1 has been observed (Häuser et al. 2015).

Fibromyalgia patients did not present apparent lesions or disease in the nervous system or evident tissue inflammation, so their condition is classified as nociplastic pain (Kosek et al. 2016). Patients develop a range of clinical symptoms, including widespread pain, evidenced by the presence of mechanical and thermal hypersensitivity and comorbid symptoms such as migraine, anxiety, depression, fatigue and sleep disturbances (Sarzi‐Puttini et al. 2020; Marcus et al. 2005). Furthermore, about one‐third of fibromyalgia patients describe their symptoms as having a neuropathic pain quality, which includes burning pain, tingling sensations, or, in some cases, dysesthesia (Häuser et al. 2017; Littlejohn and Guymer 2018). Currently, the Food and Drug Administration (FDA) and the recommend antidepressants and antiepileptics, such as duloxetine, milnacipran and pregabalin, for the treatment of fibromyalgia (Häuser et al. 2015; Macfarlane et al. 2017). However, all these drugs provide limited pain relief associated with severe adverse effects (Arnold et al. 2016), emphasising the need for innovative and mechanism‐based therapeutic approaches.

Although fibromyalgia encompasses a wide range of mechanisms across many systems, CNS involvement is a critical element (Häuser et al. 2015). Patients with fibromyalgia have increased brain‐specific pain processing (López‐Solà et al. 2017) and brain glial activation (Albrecht et al. 2019). Impaired neurotransmission function is observed, including an increase in excitatory neurotransmitters, such as glutamate and substance P, and a reduction in the inhibitory ones, including biogenic amines (dopamine, norepinephrine and epinephrine) and γ‐aminobutyric acid (GABA), which alter connectivity in the nervous system, favouring the development of central sensitisation in this syndrome (Sarzi‐Puttini et al. 2020). Other evidence suggests that central sensitisation is secondary to peripheral mechanisms in fibromyalgia, including the activation of dorsal root ganglia (DRG) by immune cells (Martínez‐Lavín 2021). In fibromyalgia patients, nociceptors have a reduced threshold to pain and other external stimuli, resulting in increased pain sensitivity (Littlejohn and Guymer 2018). Additionally, a variable proportion of patients with fibromyalgia have C‐fibre hyperexcitability (Serra et al. 2014) and reduced intraepidermal nerve fibre density (Fasolino et al. 2020; Doppler et al. 2015; Üçeyler et al. 2013). Small fibre dysfunction could explain the dysesthesia observed in some patients with fibromyalgia (Sarzi‐Puttini et al. 2020). However, the association between fibromyalgia pain severity and small fibre pathology remains inconsistently characterised (Fasolino et al. 2020; Oaklander 2016), with conflicting evidence regarding whether small fibre pathology significantly affects somatosensory system function in patients. Nevertheless, these findings support the concept of peripheral sensitisation in this syndrome.

Neurogenic inflammation has been proposed to contribute to fibromyalgia syndrome. This process involves the release of several neuropeptides, chemokines and cytokines, which activate the innate and adaptive immune systems (Littlejohn and Guymer 2018). Although peripheral monocyte counts are not increased in fibromyalgia patients compared with healthy controls (Taylor et al. 2016), elevated serum levels of macrophage markers, along with various cytokines and chemokines, suggest a possible proinflammatory signature of fibromyalgia (García et al. 2016; Tripathi et al. 2021). Furthermore, mastocytosis and subsequent activation of glial cells might contribute to neuroinflammation in the syndrome (Littlejohn and Guymer 2018; Theoharides et al. 2019; Blanco et al. 2010; Brum et al. 2024a).

Growing attention has been directed toward mitochondrial dysfunction and oxidative stress, which are now recognised as critical mechanisms underlying both the development and persistence of fibromyalgia (Meeus et al. 2013). Mitochondrial dysfunction observed in patients with fibromyalgia is characterised by structural alterations such as degenerated mitochondria with acid vacuoles and irregular cristae (Cordero et al. 2010; Park et al. 2000), as well as molecular abnormalities including mitochondrial DNA deletions, activation of apoptotic pathways (Meeus et al. 2013), mitophagy, reduced mitochondrial membrane potential, coenzyme Q10 deficiency (Cordero et al. 2010) and impaired activity of respiratory chain complexes (Sánchez‐Domínguez et al. 2015). Moreover, decreased activity of oxidative enzymes, such as citrate synthase and cytochrome oxidase, further supports defects in oxidative metabolism and impaired ATP synthesis in fibromyalgia (Meeus et al. 2013).

Recent evidence has demonstrated a strong association between pro‐oxidative processes and pain sensitisation in fibromyalgia. Patients with fibromyalgia had elevated levels of protein carbonyls (Cordero et al. 2009), nitric oxide (Shukla et al. 2020), thiobarbituric acid–reactive substances (Cordero et al. 2010), malondialdehyde (Bagis et al. 2005) and peroxides (Altindag and Celik 2006) in skin biopsies, plasma and peripheral blood mononuclear cells. Importantly, levels of lipid peroxidation products positively correlate with the severity of fibromyalgia (Hung et al. 2020). In contrast, antioxidant defences, including levels of vitamins A and E (Akkuş et al. 2009), and activity of superoxide dismutase, catalase, glutathione reductase, glutathione peroxidase and NADPH oxidase (NOX), are reduced compared to those of control patients (Shukla et al. 2020; Rus et al. 2021), and their levels are negatively associated with fibromyalgia symptoms, including overall functioning, pain, psychological distress and sleep quality (Assavarittirong et al. 2022).

The interplay between mitochondrial dysfunction and oxidative stress is thought to contribute to both peripheral and central sensitisation, thereby playing a crucial role in the development of the chronic widespread pain characteristic of fibromyalgia (Meeus et al. 2013; Brum et al. 2020). Consequently, therapeutic approaches with antioxidant properties, such as hyperbaric oxygen therapy, aerobic exercises and antioxidant supplementation with, for example, coenzyme Q10 and melatonin, have been investigated as potential strategies to alleviate fibromyalgia symptoms (Assavarittirong et al. 2022). Nevertheless, due to limitations including poor pharmacokinetics and inconsistent clinical outcomes (Al‐Madhagi and Masoud 2024), current antioxidant‐based interventions have not demonstrated sufficient efficacy to be recommended in clinical guidelines (Macfarlane et al. 2017).

Although several mechanisms underlying fibromyalgia have been described, it remains challenging to determine which of these mechanisms are truly pathogenic and which represent secondary or minor consequences of the syndrome. A deeper understanding of the pathogenic mechanisms of fibromyalgia would undoubtedly enable the development of more appropriate and effective therapies. Among potential targets, the TRPA1 channel has emerged as a key sensor of oxidative stress and a mediator of pain hypersensitivity. Targeting TRPA1‐regulated pro‐oxidant signalling pathways may therefore represent a promising therapeutic strategy, which can be effectively investigated using experimental models of fibromyalgia.

## 3 The Role of Schwann Cell TRPA1 in a Fibromyalgia‐Like Phenotype

Growing evidence has highlighted the pivotal role of Schwann cells in modulating and maintaining pain. Schwann cells form the myelin sheaths that insulate neuronal axons and support efficient neural transmission, while they also release trophic factors that nourish and protect axons (Zhang et al. 2023). In 2017, De Logu and colleagues showed that Schwann cells expressed TRPA1. Right after, Abdo and collaborators discovered a specialised cutaneous glial cell type, closely associated with unmyelinated nociceptive fibres, that detects noxious thermal and mechanical stimuli and conveys nociceptive information to the nerve (Abdo et al. 2019). These findings revealed a previously unknown function of Schwann cells. They made it possible to demonstrate that TRPA1 expressed in Schwann cells sustains hypersensitivity in different mouse models of pain.

In the peripheral nerve injury model, for example, macrophages recruited by CCL2 generate a NOX2‐dependent oxidative burst that targets TRPA1 channels in Schwann cells. In turn, NOX1 activation, via TRPA1‐mediated calcium mobilisation, induces sustained oxidative stress, which maintains macrophage infiltration into the injured nerve and ultimately activates TRPA1 on nociceptor fibres, leading to allodynia (De Logu et al. 2017). Similarly, in the ischemia/reperfusion model, the initial oxidative burst that follows reperfusion activates a feed‐forward mechanism involving resident macrophages and Schwann cell TRPA1, which sustains chronic neuroinflammation and pain (De Logu, De Prá, et al. 2020).

In alcoholic neuropathy, Schwann cells ensheathing plantar nerve fibres express the enzyme aldehyde dehydrogenase, which mediates the local conversion of ethanol into acetaldehyde. Selective deletion of TRPA1 in Schwann cells or nociceptors revealed that acetaldehyde, generated either systemically or locally, activates TRPA1 in Schwann cells, leading to NOX1‐dependent oxidative and carbonyl stress that, in turn, targets neuronal TRPA1 to sustain allodynia (De Logu et al. 2019; Landini, Souza Monteiro de Araujo, et al. 2023). In a cancer pain model, neuroinflammation, mechanical and cold hypersensitivity and spontaneous nociception are maintained by a feed‐forward mechanism that requires continuous crosstalk between Schwann cell TRPA1 and resident macrophages along the sciatic nerve trunk (De Logu et al. 2021). Finally, in a migraine model, CGRP released from cutaneous trigeminal fibres activates CGRP receptors on surrounding Schwann cells, triggering prolonged endosomal signalling via cAMP and nitric oxide production. Nitric oxide, in turn, activates TRPA1 channels in Schwann cells, resulting in ROS release that, in a feed‐forward manner, sustains periorbital mechanical allodynia via nociceptor TRPA1 (De Logu et al. 2022).

Mechanistically, these studies converge on a redox‐dependent feed‐forward signalling loop in which injurious or inflammatory stimuli generate local oxidative or carbonylic stress that activates TRPA1 expressed by Schwann cells. TRPA1 activation leads to sustained production of reactive oxygen and nitrogen species, creating a localised pro‐oxidant microenvironment that promotes reciprocal activation of macrophages and nociceptor terminals, thereby sustaining neuroinflammation and persistent hypersensitivity. Through this mechanism, Schwann cell TRPA1 extends beyond its sensory role to sustain a pro‐oxidant microenvironment that favours the persistence of chronic pain.

In fibromyalgia, the epigenetic regulation of TRPA1 or genetic variants has been associated with reduced mechanical and cold pain thresholds (D'Agnelli et al. 2019; Marchi et al. 2023). As the imbalance of oxidant and antioxidant molecules appears to play an essential role in the pathogenesis of fibromyalgia (Assavarittirong et al. 2022), it is reasonable that the ROS sensor TRPA1 may also be mediating fibromyalgia symptoms. ROS generated by different cellular sources target Schwann cell TRPA1 to amplify a ROS‐dependent feed‐forward mechanism that sustains neuroinflammation and neuropathic pain (De Logu et al. 2017; De Logu, De Prá, et al. 2020). Thus, Brum and colleagues explored the mechanisms driven by ROS and Schwann cell TRPA1 in the reserpine‐induced fibromyalgia model (Brum et al. 2024b; Brum et al. 2025). The choice of this model was based on its well‐established face, construct and predictive validities (Brum et al. 2022; Nagakura 2015; Nagakura 2022). However, although it is one of the most widely used experimental models of fibromyalgia, it has limitations, as reserpine‐induced changes in biogenic amines also play an essential role in motor control (Nagakura et al. 2009; Nagakura et al. 2018).

The reserpine administration has been associated with sensory and cognitive changes that mirror fibromyalgia (Brum et al. 2022; Nagakura 2022). The murine fibromyalgia model induced pain‐like behaviours, including plantar mechanical and cold hypersensitivity and early anxiety‐depressive‐like disorders (Brum et al. 2024b), similar to mechanical and cold allodynia described by fibromyalgia patients (Littlejohn and Guymer 2018; Larson et al. 2014), as well as reports of anxiety and depression (Choy 2015). The same fibromyalgia model also caused periorbital mechanical allodynia (Brum et al. 2025), a parameter that recapitulates one of the major symptoms of migraine, which is present in about 50% of patients with fibromyalgia (Marcus et al. 2005).

Pregabalin and duloxetine are among the FDA‐ and EULAR‐recommended drugs for fibromyalgia (Macfarlane et al. 2017; Sarzi‐Puttini et al. 2020) and are presumed to have a CNS effect (Arnold et al. 2016). Although they are only partially effective and cause adverse effects, such as weight gain, drowsiness, blurred vision, dry mouth and somnolence (Arnold et al. 2016), they are employed in preclinical research to estimate the predictive validity of fibromyalgia models (Brum et al. 2022). Taking this into consideration, pregabalin and duloxetine reduced pain‐like and depressive‐like behaviour, respectively, but did not affect the neuroinflammation parameters induced in the model (Brum et al. 2024b). This suggests that the reserpine‐induced fibromyalgia model initially involves the peripheral nervous system with a subsequent CNS involvement. Additionally, olcegepant (a CGRP receptor antagonist) reversed the reserpine‐induced periorbital mechanical allodynia (Brum et al. 2025), demonstrating the effect of a peripherally acting antimigraine drug on this experimental pain model.

Next, it was verified that there was an increase in 4‐hydroxynonenal and hydrogen peroxide levels, markers of oxidative stress, in the sciatic and trigeminal nerve tissue of mice subjected to the fibromyalgia model (Brum et al. 2024b; Brum et al. 2025). Such data are consistent with the presence of oxidative stress biomarkers in both clinical (Cordero et al. 2011; Assavarittirong et al. 2022) and preclinical (Brum et al. 2020; Favero et al. 2019) fibromyalgia settings. Either the levels of oxidative stress markers or the fibromyalgia‐like behaviours were mitigated by treatment with the antioxidant α‐lipoic acid and the ROS spin trap phenyl‐α‐tert‐butyl nitrone (PBN) (Brum et al. 2024b; Brum et al. 2025), indicating that by‐products of oxidative stress generated during the establishment of the condition contribute to these symptoms. The antioxidant α‐lipoic acid has shown preclinical efficacy in chronic pain models (Brusco et al. 2019; Dalenogare et al. 2020; Peres et al. 2021) and has shown preliminary efficacy in patients with fibromyalgia (Gilron et al. 2017a, 2017b; Vittorio et al. 2020). However, given the limited clinical benefit of antioxidant therapies (Salehi et al. 2019), identifying specific downstream targets of ROS signalling has become increasingly important.

The fibromyalgia model facilitated the accumulation of macrophages within peripheral sciatic and trigeminal nerve tissue (Brum et al. 2024b; Brum et al. 2025), similar to that observed after partial sciatic nerve ligation (De Logu et al. 2017), ischemia/reperfusion (De Logu, De Prá, et al. 2020) and cancer pain (De Logu et al. 2021). Although to date, few studies have reported increased macrophage numbers in the reserpine‐experimental model, these results align with findings that the recruitment and activation of innate immune cells contribute to neuroinflammation in patients with fibromyalgia (Littlejohn and Guymer 2018; Theoharides et al. 2019). In Macrophage Fas‐Induced Apoptosis (MaFIA) mice, which exhibit a remarkable pharmacologically produced depletion of macrophages (Shepherd et al. 2018), the fibromyalgia model induced by reserpine did not cause neuroinflammation, plantar mechanical and cold hypersensitivity, periorbital mechanical allodynia, or early anxiety‐depressive‐like behaviours (Brum et al. 2024b; Brum et al. 2025). Activated macrophages serve as a significant source of the oxidative burst, and, just as they have been shown to support neuroinflammation and pain in other murine models (De Logu et al. 2017; De Logu, De Prá, et al. 2020), they also appear to play a crucial role in patients with fibromyalgia (Tripathi et al. 2021).

Later, it was observed that selective pharmacological antagonism of TRPA1 (with A967079) or global knockout mitigated fibromyalgia‐like behaviours. When the fibromyalgia model was induced in *Trpa1* knockout mice, the number of intrasciatic and intratrigeminal F4/80^+^ cells (a murine marker of macrophages) and the levels of 4‐hydroxynonenal did not increase, indicating that both neuroinflammation and fibromyalgia‐like behaviours depend on TRPA1. However, since TRPA1 has important physiological functions, ranging from nociception and temperature sensation to homeostatic functions, muscle contraction and vasomotor control (Maglie et al. 2021), overall inhibition is not recommended. Independently, just as observed in neuropathic and cancer pain models (De Logu et al. 2017; De Logu et al. 2019; De Logu et al. 2021), the TRPA1 channel expressed exclusively in sensory neurons mediated the fibromyalgia‐like behaviours, while the TRPA1 in Schwann cells was responsible for eliciting not only the fibromyalgia‐like behaviours but also the neuroinflammation (Brum et al. 2024b, 2025).

Additionally, a series of studies has reported that the activation of Schwann cell TRPA1 results in the release of ROS, which induces a calcium‐dependent activation of NOX1 that amplifies a feedforward mechanism to sustain neuroinflammation (De Logu et al. 2017; De Logu, De Prá, et al. 2020; Landini, Souza Monteiro de Araujo, et al. 2023; De Logu et al. 2022). When NOX1 was pharmacologically inhibited (using both non‐selective and selective inhibitors, apocynin and ML‐171, respectively), neuroinflammation and fibromyalgia‐like behaviours were reduced (Brum et al. 2024b, 2025). Thus, the findings further support the hypothesis that TRPA1/NOX1 interaction is a plausible pathway for generating oxidative stress that sustains fibromyalgia‐like behaviours. Nevertheless, selectively targeting the pharmacological inhibition of these proteins within this specific cell type remains a substantial translational challenge.

Despite the strong mechanistic evidence linking oxidative stress, Schwann cell TRPA1, and fibromyalgia‐like behaviours, several limitations should be acknowledged. The reserpine‐induced model, while exhibiting robust face, construct and predictive validity, does not fully capture the clinical heterogeneity and multifactorial nature of fibromyalgia. Moreover, the proposed TRPA1/NOX1 redox feed‐forward mechanism is primarily supported by preclinical data, and species‐specific differences may limit direct translation to human disease. The global genetic deletion and systemic pharmacological inhibition also precludes complete definition of cell–type–specific contributions, and the lack of selective Schwann cell–targeted interventions remains a translational challenge. Furthermore, it is unclear whether this peripheral mechanism predominates throughout disease progression or represents an early pathogenic driver that later converges with central sensitisation processes.

## 4 Conclusion

TRPA1 has been proposed to play a substantial role in pain perception, acting as a sensor of environmental changes (Maglie et al. 2021; Andrade et al. 2012). In addition to detecting mechanical and thermal stimuli, it also senses and amplifies oxidative stress (Mori et al. 2016). Studies have evidenced the association between TRPA1 and reduced mechanical and cold pain thresholds in fibromyalgia patients (Achenbach et al. 2019). Data from Brum and collaborators indicate that activated Schwann cells expressing TRPA1 initiate an intracellular signalling pathway culminating in ROS release and macrophage recruitment in the mouse sciatic and trigeminal nerves. These mechanisms contribute to mechanical and cold hypersensitivity, as well as early anxiety‐ and depression‐like behaviours in the reserpine‐induced fibromyalgia model (Brum et al. 2024b, 2025). Since TRPA1 has important physiological functions (Maglie et al. 2021; Andrade et al. 2012), overall inhibition is not recommended. However, if this model reliably recapitulates the features of the human disease, targeting TRPA1 channels on Schwann cells could represent a promising therapeutic approach for managing fibromyalgia‐related symptoms, ultimately improving patients' quality of life and their relationship to the disease. Future translational studies will be essential to validate whether pharmacological modulation of the Schwann cell TRPA1/NOX1 pathway can provide clinical benefit in fibromyalgia, bridging the gap between preclinical findings and therapeutic application.

## Author Contributions

Study concept and design: E.S.B., S.M.O. Drafting and revising the content of the manuscript: E.S.B., M.F.P.F., D.S.M.A., L.L., M.M., F.D.L., R.N. and S.M.O.

## Funding

This study was supported by the Coordenação de Aperfeiçoamento de Pessoal de Nível Superior—Brasil (CAPES), Finance Code 001; the CAPES/Programa de Excelencia Academica (PROEX) (process #88881.844988/2023–01; AUXPE #1333/2023) and by the Fundação de Amparo à Pesquisa do Estado do Rio Grande do Sul (FAPERGS; Grant #25/2551‐0000849‐2, 10/2024 ARD/ARC, E.S.B.). S.M.O. is a recipient of a grant from Conselho Nacional de Desenvolvimento Científico e Tecnológico (CNPq) (Grant #309404/2023–1). E.S.B. was a recipient of fellowships from CNPq (#150611/2022‐6), CAPES/PROEX (#88887.185973/2018–00) and Programa Institucional de Internacionalizaçao from CAPES (CAPES PrInt) (#88887.475201/2020–00)., F.D.L. is a recipient of a grant from Fondo Italiano per la Scienza 2022‐2023 (FIS‐2023‐03323). We thank the Federal University of Rio Grande do Sul, the Federal University of Santa Maria, the Fluminense Federal University, New York University, and the University of Florence.

## Conflicts of Interest

All the authors declare no conflicts of interest.

## Data Availability

The authors have nothing to report.

## References

[jnc70424-bib-0001] Abdo, H. , L. Calvo‐Enrique , J. M. Lopez , et al. 2019. “Specialized Cutaneous Schwann Cells Initiate Pain Sensation.” Science 365: 695–699. 31416963 10.1126/science.aax6452

[jnc70424-bib-0002] Achenbach, J. , M. Rhein , S. Gombert , et al. 2019. “Childhood Traumatization Is Associated With Differences in TRPA1 Promoter Methylation in Female Patients With Multisomatoform Disorder With Pain as the Leading Bodily Symptom.” Clinical Epigenetics 11: 1–10. 31455424 10.1186/s13148-019-0731-0 PMC6712620

[jnc70424-bib-0003] Akkuş, S. , M. Naziroǧlu , S. Eriş , K. Yalman , N. Yilmaz , and M. Yener . 2009. “Levels of Lipid Peroxidation, Nitric Oxide, and Antioxidant Vitamins in Plasma of Patients With Fibromyalgia.” Cell Biochemistry and Function 27: 181–185. 19319826 10.1002/cbf.1548

[jnc70424-bib-0004] Albrecht, D. S. , A. Forsberg , A. Sandström , et al. 2019. “Brain Glial Activation in Fibromyalgia—A Multi‐Site Positron Emission Tomography Investigation.” Brain, Behavior, and Immunity 75: 72–83. 30223011 10.1016/j.bbi.2018.09.018 PMC6541932

[jnc70424-bib-0005] Al‐Madhagi, H. , and A. Masoud . 2024. “Limitations and Challenges of Antioxidant Therapy.” Phytotherapy Research 38: 5549–5566. 39260385 10.1002/ptr.8335

[jnc70424-bib-0006] Altindag, O. , and H. Celik . 2006. “Total Antioxidant Capacity and the Severity of the Pain in Patients With Fibromyalgia.” Redox Report 11: 131–135. 16805968 10.1179/135100006X116628

[jnc70424-bib-0007] Andersson, D. A. , C. Gentry , S. Moss , and S. Bevan . 2008. “Transient Receptor Potential A1 Is a Sensory Receptor for Multiple Products of Oxidative Stress.” Journal of Neuroscience 28: 2485–2494. 18322093 10.1523/JNEUROSCI.5369-07.2008 PMC2709206

[jnc70424-bib-0008] Andrade, E. L. , F. C. Meotti , and J. B. Calixto . 2012. “TRPA1 Antagonists as Potential Analgesic Drugs.” Pharmacology & Therapeutics 133: 189–204. 22119554 10.1016/j.pharmthera.2011.10.008

[jnc70424-bib-0009] Antoniazzi, C. T. D. D. , R. Nassini , F. K. Rigo , et al. 2019. “Transient Receptor Potential Ankyrin 1 (TRPA1) Plays a Critical Role in a Mouse Model of Cancer Pain.” International Journal of Cancer 144: 355–365. 30289972 10.1002/ijc.31911 PMC6587729

[jnc70424-bib-0010] Arnold, L. M. , R. M. Bennett , L. J. Crofford , et al. 2019. “AAPT Diagnostic Criteria for Fibromyalgia.” Journal of Pain 20: 611–628. 30453109 10.1016/j.jpain.2018.10.008

[jnc70424-bib-0011] Arnold, L. M. , K. B. Gebke , and E. H. S. Choy . 2016. “Fibromyalgia: Management Strategies for Primary Care Providers.” International Journal of Clinical Practice 70: 99–112. 26817567 10.1111/ijcp.12757 PMC6093261

[jnc70424-bib-0012] Assavarittirong, C. , W. Samborski , and B. Grygiel‐Górniak . 2022. “Oxidative Stress in Fibromyalgia: From Pathology to Treatment.” Oxidative Medicine and Cellular Longevity 2022: 1–11. 10.1155/2022/1582432 PMC9556195 36246401

[jnc70424-bib-0013] Atoyan, R. , D. Shander , and N. V. Botchkareva . 2009. “Non‐Neuronal Expression of Transient Receptor Potential Type A1 (TRPA1) in Human Skin.” Journal of Investigative Dermatology 129: 2312–2315. 19282836 10.1038/jid.2009.58

[jnc70424-bib-0014] Bagis, S. , L. Tamer , G. Sahin , et al. 2005. “Free Radicals and Antioxidants in Primary Fibromyalgia: An Oxidative Stress Disorder?” Rheumatology International 25: 188–190. 14689230 10.1007/s00296-003-0427-8

[jnc70424-bib-0015] Becker, G. , M. F. P. Fialho , E. S. Brum , and S. M. Oliveira . 2023. “Kinin B2 Receptor Mediates Cisplatin‐Induced Painful Peripheral Neuropathy by Intracellular Kinase Pathways and TRPA1 Channel Sensitisation.” Pharmaceuticals 16: 959. 37513871 10.3390/ph16070959 PMC10386204

[jnc70424-bib-0016] Blanco, I. , N. Béritze , M. Argüelles , et al. 2010. “Abnormal Overexpression of Mastocytes in Skin Biopsies of Fibromyalgia Patients.” Clinical Rheumatology 29: 1403–1412. 20428906 10.1007/s10067-010-1474-7

[jnc70424-bib-0017] Brum, E. d. S. , M. F. P. Fialho , G. Becker , C. W. Nogueira , and S. M. Oliveira . 2024a. “Involvement of Peripheral Mast Cells in a Fibromyalgia Model in Mice.” European Journal of Pharmacology 967: 176385. 38311276 10.1016/j.ejphar.2024.176385

[jnc70424-bib-0018] Brum, E. d. S. , M. F. P. Fialho , S. P. M. Fischer , et al. 2020. “Relevance of Mitochondrial Dysfunction in the Reserpine‐Induced Experimental Fibromyalgia Model.” Molecular Neurobiology 57: 4202–4217. 32685997 10.1007/s12035-020-01996-1

[jnc70424-bib-0019] Brum, E. S. , G. Becker , M. F. P. Fialho , and S. M. Oliveira . 2022. “Animal Models of Fibromyalgia: What Is the Best Choice?” Pharmacology & Therapeutics 230: 107959. 34265360 10.1016/j.pharmthera.2021.107959

[jnc70424-bib-0020] Brum, E. S. , M. F. P. Fialho , D. Souza Monteiro de Araújo , et al. 2024b. “Schwann Cell TRPA1 Elicits Reserpine‐Induced Fibromyalgia Pain in Mice.” British Journal of Pharmacology 181: 3445–3461. 38772415 10.1111/bph.16413

[jnc70424-bib-0021] Brum, E. S. , L. Landini , D. Souza Monteiro de Araújo , et al. 2025. “Characterisation of Periorbital Mechanical Allodynia in the Reserpine‐Induced Fibromyalgia Model in Mice: The Role of the Schwann Cell TRPA1/NOX1 Signalling Pathway.” Free Radical Biology and Medicine 229: 289–299. 39842732 10.1016/j.freeradbiomed.2025.01.040

[jnc70424-bib-0022] Brusco, I. , S. Li Puma , K. B. Chiepe , et al. 2019. “Dacarbazine Alone or Associated With Melanoma‐Bearing Cancer Pain Model Induces Painful Hypersensitivity by TRPA1 Activation in Mice.” International Journal of Cancer 293: 1–14. 10.1002/ijc.32648 31456221

[jnc70424-bib-0023] Choy, E. H. S. 2015. “The Role of Sleep in Pain and Fibromyalgia.” Nature Reviews Rheumatology 11: 513–520. 25907704 10.1038/nrrheum.2015.56

[jnc70424-bib-0024] Clauw, D. J. 2014. “Fibromyalgia: A Clinical Review.” JAMA 311: 1547–1555. 24737367 10.1001/jama.2014.3266

[jnc70424-bib-0025] Cordero, M. D. , E. Alcocer‐Gómez , F. J. Cano‐García , et al. 2011. “Clinical Symptoms in Fibromyalgia Are Better Associated to Lipid Peroxidation Levels in Blood Mononuclear Cells Rather Than in Plasma.” PLoS One 6: e26915. 22046409 10.1371/journal.pone.0026915 PMC3203929

[jnc70424-bib-0026] Cordero, M. D. , M. de Miguel , A. M. Moreno Fernández , et al. 2010. “Mitochondrial Dysfunction and Mitophagy Activation in Blood Mononuclear Cells of Fibromyalgia Patients: Implications in the Pathogenesis of the Disease.” Arthritis Research & Therapy 12: R17. 20109177 10.1186/ar2918 PMC2875645

[jnc70424-bib-0027] Cordero, M. D. , A. M. Moreno‐Fernández , M. deMiguel , et al. 2009. “Coenzyme Q10 Distribution in Blood Is Altered in Patients With Fibromyalgia.” Clinical Biochemistry 42: 732–735. 19133251 10.1016/j.clinbiochem.2008.12.010

[jnc70424-bib-0028] da Costa, D. S. M. , F. C. Meotti , E. L. Andrade , P. C. Leal , E. M. Motta , and J. B. Calixto . 2010. “The Involvement of the Transient Receptor Potential A1 (TRPA1) in the Maintenance of Mechanical and Cold Hyperalgesia in Persistent Inflammation.” Pain 148: 431–437. 20056530 10.1016/j.pain.2009.12.002

[jnc70424-bib-0029] D'Agnelli, S. , L. Arendt‐Nielsen , M. C. Gerra , et al. 2019. “Fibromyalgia: Genetics and Epigenetics Insights May Provide the Basis for the Development of Diagnostic Biomarkers.” Molecular Pain 15: 1744806918819944. 30486733 10.1177/1744806918819944 PMC6322092

[jnc70424-bib-0030] Dalenogare, D. P. , M. C. Theisen , D. S. Peres , et al. 2020. “TRPA1 Activation Mediates Nociception Behaviors in a Mouse Model of Relapsing‐Remitting Experimental Autoimmune Encephalomyelitis.” Experimental Neurology 328: 113241. 32045597 10.1016/j.expneurol.2020.113241

[jnc70424-bib-0031] de David Antoniazzi, C. T. , S. D. T. De Prá , P. R. Ferro , et al. 2018. “Topical Treatment With a Transient Receptor Potential Ankyrin 1 (TRPA1) Antagonist Reduced Nociception and Inflammation in a Thermal Lesion Model in Rats.” European Journal of Pharmaceutical Sciences 125: 28–38. 30236550 10.1016/j.ejps.2018.09.012

[jnc70424-bib-0032] De Logu, F. , S. D. T. De Prá , C. T. de David Antoniazzi , et al. 2020. “Macrophages and Schwann Cell TRPA1 Mediate Chronic Allodynia in a Mouse Model of Complex Regional Pain Syndrome Type I.” Brain, Behavior, and Immunity 88: 535–546. 32315759 10.1016/j.bbi.2020.04.037

[jnc70424-bib-0033] De Logu, F. , M. Marini , L. Landini , et al. 2021. “Peripheral Nerve Resident Macrophages and Schwann Cells Mediate Cancer‐Induced Pain.” Cancer Research 81: 3387–3401. 33771895 10.1158/0008-5472.CAN-20-3326 PMC8260461

[jnc70424-bib-0034] De Logu, F. , R. Nassini , A. Hegron , et al. 2022. “Schwann Cell Endosome CGRP Signals Elicit Periorbital Mechanical Allodynia in Mice.” Nature Communications 13: 646. 10.1038/s41467-022-28204-z PMC8813987 35115501

[jnc70424-bib-0035] De Logu, F. , R. Nassini , S. Materazzi , et al. 2017. “Schwann Cell TRPA1 Mediates Neuroinflammation That Sustains Macrophage‐Dependent Neuropathic Pain in Mice.” Nature Communications 8: 1–16. 10.1038/s41467-017-01739-2 PMC5709495 29192190

[jnc70424-bib-0036] De Logu, F. , S. L. Puma , L. Landini , et al. 2019. “Schwann Cells Expressing Nociceptive Channel TRPA1 Orchestrate Ethanol‐Evoked Neuropathic Pain in Mice.” Journal of Clinical Investigation 129: 5424–5441. 31487269 10.1172/JCI128022 PMC6877331

[jnc70424-bib-0037] De Logu, F. , R. Tonello , S. Materazzi , et al. 2016. “TRPA1 Mediates Aromatase Inhibitor–Evoked Pain by the Aromatase Substrate Androstenedione.” Cancer Research 76: 7024–7035. 27758889 10.1158/0008-5472.CAN-16-1492

[jnc70424-bib-0038] De Logu, F. , G. Trevisan , I. M. Marone , et al. 2020. “Oxidative Stress Mediates Thalidomide‐Induced Pain by Targeting Peripheral TRPA1 and Central TRPV4.” BMC Biology 18: 197. 33317522 10.1186/s12915-020-00935-9 PMC7737339

[jnc70424-bib-0039] Doppler, K. , H. L. Rittner , M. Deckart , and C. Sommer . 2015. “Reduced Dermal Nerve Fiber Diameter in Skin Biopsies of Patients With Fibromyalgia.” Pain 156: 2319–2325. 26164586 10.1097/j.pain.0000000000000285

[jnc70424-bib-0040] Fasolino, A. , G. di Stefano , C. Leone , et al. 2020. “Small‐Fibre Pathology Has no Impact on Somatosensory System Function in Patients With Fibromyalgia.” Pain 161: 2385–2393. 32897040 10.1097/j.pain.0000000000001920

[jnc70424-bib-0041] Favero, G. , F. Bonomini , C. Franco , and R. Rezzani . 2019. “Mitochondrial Dysfunction in Skeletal Muscle of a Fibromyalgia Model: The Potential Benefits of Melatonin.” International Journal of Molecular Sciences 20: 765. 30754674 10.3390/ijms20030765 PMC6386947

[jnc70424-bib-0042] Fialho, M. F. P. , E. S. Brum , G. Becker , I. Brusco , and S. M. Oliveira . 2023. “Kinin B2 and B1 Receptors Activation Sensitize the TRPA1 Channel Contributing to Anastrozole‐Induced Pain Symptoms.” Pharmaceutics 15: 1136. 37111622 10.3390/pharmaceutics15041136 PMC10143169

[jnc70424-bib-0043] Forman, H. J. , and H. Zhang . 2021. “Targeting Oxidative Stress in Disease: Promise and Limitations of Antioxidant Therapy.” Nature Reviews. Drug Discovery 20: 689–709. 34194012 10.1038/s41573-021-00233-1 PMC8243062

[jnc70424-bib-0044] Fusi, C. , S. Materazzi , S. Benemei , et al. 2014. “Steroidal and Non‐Steroidal Third‐Generation Aromatase Inhibitors Induce Pain‐Like Symptoms via TRPA1.” Nature Communications 5: 1–14. 10.1038/ncomms6736 PMC4268712 25484020

[jnc70424-bib-0045] García, J. J. , J. Carvajal‐Gil , and R. Guerrero‐Bonmatty . 2016. “Altered Release of Chemokines by Phagocytes From Fibromyalgia Patients: A Pilot Study.” Innate Immunity 22: 3–8. 26341115 10.1177/1753425915602959

[jnc70424-bib-0046] Gilron, I. , D. Tu , R. Holden , T. Towheed , E. Vandenkerkhof , and R. Milev . 2017a. “Combination Analgesic Development for Enhanced Clinical Efficacy (CADENCE Trial): Study Protocol for a Double‐Blind, Randomized, Placebo‐Controlled Crossover Trial of an Alpha‐Lipoic Acid—Pregabalin Combination for the Treatment of Fibromyalgia Pain.” JMIR Research Protocols 6: e154. 28778847 10.2196/resprot.8001 PMC5705061

[jnc70424-bib-0047] Gilron, I. , D. Tu , R. Holden , et al. 2017b. “Innovations in the Management of Musculoskeletal Pain With Alpha‐Lipoic Acid (IMPALA Trial): Study Protocol for a Double‐Blind, Randomized, Placebo‐Controlled Crossover Trial of Alpha‐Lipoic Acid for the Treatment of Fibromyalgia Pain.” JMIR Research Protocols 6: e41. 28351829 10.2196/resprot.7198 PMC5388826

[jnc70424-bib-0048] Hamilton, N. B. , K. Kolodziejczyk , E. Kougioumtzidou , and D. Attwell . 2016. “Proton‐Gated Ca^2+^−Permeable TRP Channels Damage Myelin in Conditions Mimicking Ischaemia.” Nature 529: 523–527. 26760212 10.1038/nature16519 PMC4733665

[jnc70424-bib-0049] Häuser, W. , J. Ablin , M. A. Fitzcharles , et al. 2015. “Fibromyalgia.” Nature Reviews Disease Primers 1: 1–16. 10.1038/nrdp.2015.22 27189527

[jnc70424-bib-0050] Häuser, W. , S. Perrot , C. Sommer , Y. Shir , and M. Fitzcharles . 2017. “Diagnostic Confounders of Chronic Widespread Pain: Not Always Fibromyalgia.” PAIN Reports 2: 1–11. 10.1097/PR9.0000000000000598 PMC5741304 29392213

[jnc70424-bib-0051] Hung, C. H. , C. H. Lee , M. H. Tsai , et al. 2020. “Activation of Acid‐Sensing Ion Channel 3 by Lysophosphatidylcholine 16:0 Mediates Psychological Stress‐Induced Fibromyalgia‐Like Pain.” Annals of the Rheumatic Diseases 79: 1644–1656. 32907805 10.1136/annrheumdis-2020-218329 PMC7677496

[jnc70424-bib-0052] Jaquemar, D. , T. Schenker , and B. Trueb . 1999. “An Ankyrin‐Like Protein With Transmembrane Domains Is Specifically Lost After Oncogenic Transformation of Human Fibroblasts *.” Biochemistry 274: 7325–7333. 10.1074/jbc.274.11.7325 10066796

[jnc70424-bib-0053] Kochukov, M. Y. , T. A. McNearney , Y. Fu , and K. N. Westlund . 2006. “Thermosensitive TRP Ion Channels Mediate Cytosolic Calcium Response in Human Synoviocytes.” American Journal of Physiology. Cell Physiology 291: C424–C432. 16597917 10.1152/ajpcell.00553.2005

[jnc70424-bib-0054] Koivisto, A. , and A. Pertovaara . 2013. “Transient Receptor Potential Ankyrin 1 (TRPA1) Ion Channel in the Pathophysiology of Peripheral Diabetic Neuropathy.” Scandinavian Journal of Pain 4: 129–136. 29913916 10.1016/j.sjpain.2012.11.001

[jnc70424-bib-0055] Kosek, E. , M. Cohen , R. Baron , et al. 2016. “Do We Need a Third Mechanistic Descriptor for Chronic Pain States?” Pain 157: 1382–1386. 26835783 10.1097/j.pain.0000000000000507

[jnc70424-bib-0056] Kudsi, S. Q. , C. T. de David Antoniazzi , C. Camponogara , et al. 2023. “Topical Application of a TRPA1 Antagonist Reduced Nociception and Inflammation in a Model of Traumatic Muscle Injury in Rats.” Inflammopharmacology 31: 3153–3166. 37752305 10.1007/s10787-023-01337-3

[jnc70424-bib-0057] Landini, L. , D. S. M. de Araujo , M. Titiz , P. Geppetti , R. Nassini , and F. de Logu . 2022. “TRPA1 Role in Inflammatory Disorders: What Is Known So Far?” International Journal of Molecular Sciences 23: 4529. 35562920 10.3390/ijms23094529 PMC9101260

[jnc70424-bib-0058] Landini, L. , M. Marini , D. Souza Monteiro de Araujo , et al. 2023. “Schwann Cell Insulin‐Like Growth Factor Receptor Type‐1 Mediates Metastatic Bone Cancer Pain in Mice.” Brain, Behavior, and Immunity 110: 348–364. 36940752 10.1016/j.bbi.2023.03.013

[jnc70424-bib-0059] Landini, L. , D. Souza Monteiro de Araujo , M. Chieca , et al. 2023. “Acetaldehyde via CGRP Receptor and TRPA1 in Schwann Cells Mediates Ethanol‐Evoked Periorbital Mechanical Allodynia in Mice: Relevance for Migraine.” Journal of Biomedical Science 30: 28. 37101198 10.1186/s12929-023-00922-6 PMC10131321

[jnc70424-bib-0060] Larson, A. A. , J. V. Pardo , and J. D. Pasley . 2014. “Review of Overlap Between Thermoregulation and Pain Modulation in Fibromyalgia.” Clinical Journal of Pain 30: 544–555. 23887348 10.1097/AJP.0b013e3182a0e383 PMC3864605

[jnc70424-bib-0061] Littlejohn, G. , and E. Guymer . 2018. “Neurogenic Inflammation in Fibromyalgia.” Seminars in Immunopathology 40: 291–300. 29556959 10.1007/s00281-018-0672-2

[jnc70424-bib-0062] López‐Solà, M. , C.‐W. Woo , J. Pujol , et al. 2017. “Towards a Neurophysiological Signature for Fibromyalgia.” Pain 158: 34–47. 27583567 10.1097/j.pain.0000000000000707 PMC5161739

[jnc70424-bib-0063] Macfarlane, G. J. , C. Kronisch , L. E. Dean , et al. 2017. “EULAR Revised Recommendations for the Management of Fibromyalgia.” Annals of the Rheumatic Diseases 76: 318–328. 27377815 10.1136/annrheumdis-2016-209724

[jnc70424-bib-0064] Maglie, R. , D. Souza Monteiro de Araujo , E. Antiga , P. Geppetti , R. Nassini , and F. de Logu . 2021. “The Role of TRPA1 in Skin Physiology and Pathology.” International Journal of Molecular Sciences 22: 3065. 33802836 10.3390/ijms22063065 PMC8002674

[jnc70424-bib-0065] Maqboul, A. , and B. Elsadek . 2017. “A Novel Model of Cancer‐Induced Peripheral Neuropathy and the Role of TRPA1 in Pain Transduction.” Pain Research & Management 2017: 1–12. 10.1155/2017/3517207 PMC6232795 30510606

[jnc70424-bib-0066] Marchi, M. , E. Salvi , M. Andelic , et al. 2023. “TRPA1 Rare Variants in Chronic Neuropathic and Nociplastic Pain Patients.” Pain 164: 2048–2059. 37079850 10.1097/j.pain.0000000000002905 PMC10443199

[jnc70424-bib-0067] Marcus, D. A. , C. Bernstein , and T. E. Rudy . 2005. “Fibromyalgia and Headache: An Epidemiological Study Supporting Migraine as Part of the Fibromyalgia Syndrome.” Clinical Rheumatology 24: 595–601. 15902517 10.1007/s10067-005-1121-x

[jnc70424-bib-0068] Martínez‐Lavín, M. 2021. “Dorsal Root Ganglia: Fibromyalgia Pain Factory?” Clinical Rheumatology 40: 783–787. 33409721 10.1007/s10067-020-05528-z PMC7787228

[jnc70424-bib-0069] Materazzi, S. , C. Fusi , S. Benemei , P. Pedretti , R. Patacchini , and B. Nilius . 2012. “TRPA1 and TRPV4 Mediate Paclitaxel‐Induced Peripheral Neuropathy in Mice via a Glutathione‐Sensitive Mechanism.” Pflügers Archiv ‐ European Journal of Physiology 463: 561–569. 22258694 10.1007/s00424-011-1071-x

[jnc70424-bib-0070] Meeus, M. , J. Nijs , L. Hermans , D. Goubert , and P. Calders . 2013. “The Role of Mitochondrial Dysfunctions due to Oxidative and Nitrosative Stress in the Chronic Pain or Chronic Fatigue Syndromes and Fibromyalgia Patients: Peripheral and Central Mechanisms as Therapeutic Targets?” Expert Opinion on Therapeutic Targets 17: 1081–1089. 23834645 10.1517/14728222.2013.818657

[jnc70424-bib-0071] Moilanen, L. J. , M. Hämäläinen , E. Nummenmaa , et al. 2015. “Monosodium Iodoacetate‐Induced Inflammation and Joint Pain Are Reduced in TRPA1 Deficient Mice—Potential Role of TRPA1 in Osteoarthritis.” Osteoarthritis and Cartilage 23: 2017–2026. 26521748 10.1016/j.joca.2015.09.008

[jnc70424-bib-0072] Moparthi, L. , T. I. Kichko , M. Eberhardt , et al. 2016. “Human TRPA1 Is a Heat Sensor Displaying Intrinsic U‐Shaped Thermosensitivity.” Scientific Reports 6: 1–10. 27349477 10.1038/srep28763 PMC4923899

[jnc70424-bib-0073] Mori, Y. , N. Takahashi , O. K. Polat , T. Kurokawa , N. Takeda , and M. Inoue . 2016. “Redox‐Sensitive Transient Receptor Potential Channels in Oxygen Sensing and Adaptation.” Pflügers Archiv / European Journal of Physiology 468: 85–97. 26149285 10.1007/s00424-015-1716-2 PMC4700073

[jnc70424-bib-0074] Nagakura, Y. 2015. “Recent Advancements in Animal Models of Fibromyalgia.” Myopain 23: 104–111.

[jnc70424-bib-0075] Nagakura, Y. 2022. “Therapeutic Approaches to Nociplastic Pain Based on Findings in the Reserpine‐Induced Fibromyalgia‐Like Animal Model.” Journal of Pharmacology and Experimental Therapeutics 381: 106–119. 35246482 10.1124/jpet.121.001051

[jnc70424-bib-0076] Nagakura, Y. , T. Oe , T. Aoki , and N. Matsuoka . 2009. “Biogenic Amine Depletion Causes Chronic Muscular Pain and Tactile Allodynia Accompanied by Depression: A Putative Animal Model of Fibromyalgia.” Pain 146: 26–33. 19646816 10.1016/j.pain.2009.05.024

[jnc70424-bib-0077] Nagakura, Y. , N. Ohsaka , R. Azuma , et al. 2018. “Monoamine System Disruption Induces Functional Somatic Syndromes Associated Symptomatology in Mice.” Physiology & Behavior 194: 505–514. 29981307 10.1016/j.physbeh.2018.07.007

[jnc70424-bib-0078] Nakao, A. , K. Liu , N. Takahashi , and Y. Mori . 2024. “Universal Roles of the TRPA1 Channel in Oxygen‐Sensing.” Japanese Journal of Pharmacology 159: 23086. 10.1254/fpj.23086 38692881

[jnc70424-bib-0079] Nassini, R. , M. Gees , S. Harrison , et al. 2011. “Oxaliplatin Elicits Mechanical and Cold Allodynia in Rodents via TRPA1 Receptor Stimulation.” Pain 152: 1621–1631. 21481532 10.1016/j.pain.2011.02.051

[jnc70424-bib-0080] Oaklander, A. L. 2016. “What Is the Meaning of “Small‐Fiber Polyneuropathy” in Fibromyalgia? An Alternate Answer.” Pain 157: 1366–1367. 27183448 10.1097/j.pain.0000000000000526 PMC5845259

[jnc70424-bib-0081] Oh, M.‐H. , S. Y. Oh , J. Lu , et al. 2013. “TRPA1‐Dependent Pruritus in IL‐13–Induced Chronic Atopic Dermatitis.” Journal of Immunology 191: 5371–5382. 10.4049/jimmunol.1300300 PMC4175413 24140646

[jnc70424-bib-0082] Park, J. H. , K. J. Niermann , and N. Olsen . 2000. “Evidence for Metabolic Abnormalities in the Muscles of Patients With Fibromyalgia.” Current Rheumatology Reports 2: 131–140. 11123050 10.1007/s11926-000-0053-3

[jnc70424-bib-0083] Paulsen, C. E. , J. P. Armache , Y. Gao , Y. Cheng , and D. Julius . 2015. “Structure of the TRPA1 Ion Channel Suggests Regulatory Mechanisms.” Nature 520: 511–517. 25855297 10.1038/nature14367 PMC4409540

[jnc70424-bib-0084] Peres, D. S. , M. C. Theisen , M. F. P. Fialho , et al. 2021. “TRPA1 Involvement in Depression‐ and Anxiety‐Like Behaviors in a Progressive Multiple Sclerosis Model in Mice.” Brain Research Bulletin 175: 1–15. 34280479 10.1016/j.brainresbull.2021.07.011

[jnc70424-bib-0085] Rajagopal, S. , and M. Ponnusamy . 2017. “Regulatory Action of Calcium in Pain Pathway.” Frontiers in Cellular Neuroscience 16: 31–42. 10.3389/fncel.2022.928457 PMC9423735 36045899

[jnc70424-bib-0086] Rodrigues, P. , J. M. Frare , N. A. Ruviaro , et al. 2025. “Advanced Oxidation Protein Products Activated TRPA1 in a Neuropathic Multiple Sclerosis Model.” Brain 139: 16–17. 10.1093/brain/awaf306 40819275

[jnc70424-bib-0087] Rus, A. , I. Robles‐Fernandez , L. J. Martinez‐Gonzalez , R. Carmona , and M. J. Alvarez‐Cubero . 2021. “Influence of Oxidative Stress‐Related Genes on Susceptibility to Fibromyalgia.” Nursing Research 70: 44–50. 32991532 10.1097/NNR.0000000000000480

[jnc70424-bib-0088] Salehi, B. , Y. B. Yılmaz , G. Antika , et al. 2019. “Insights on the Use of α‐Lipoic Acid for Therapeutic Purposes.” Biomolecules 9: 356. 31405030 10.3390/biom9080356 PMC6723188

[jnc70424-bib-0089] Sánchez‐Domínguez, B. , P. Bullón , L. Román‐Malo , et al. 2015. “Oxidative Stress, Mitochondrial Dysfunction and, Inflammation Common Events in Skin of Patients With Fibromyalgia.” Mitochondrion 21: 69–75. 25662535 10.1016/j.mito.2015.01.010

[jnc70424-bib-0090] Sarzi‐Puttini, P. , V. Giorgi , D. Marotto , and F. Atzeni . 2020. Fibromyalgia: An Update on Clinical Characteristics, Aetiopathogenesis and Treatment. Springer US. 10.1038/s41584-020-00506-w 33024295

[jnc70424-bib-0091] Serra, J. , A. Collado , R. Solà , et al. 2014. “Hyperexcitable C Nociceptors in Fibromyalgia.” Annals of Neurology 75: 196–208. 24243538 10.1002/ana.24065

[jnc70424-bib-0092] Shepherd, A. J. , B. A. Copits , A. D. Mickle , et al. 2018. “Angiotensin II Triggers Peripheral Macrophage‐To‐Sensory Neuron Redox Crosstalk to Elicit Pain.” Journal of Neuroscience 38: 7032–7057. 29976627 10.1523/JNEUROSCI.3542-17.2018 PMC6083458

[jnc70424-bib-0093] Shukla, V. , S. K. Das , A. A. Mahdi , S. Agarwal , and S. Khandpur . 2020. “Nitric Oxide, Lipid Peroxidation Products, and Antioxidants in Primary Fibromyalgia and Correlation With Disease Severity.” Journal of Medical Biochemistry 39: 165–170. 33033448 10.2478/jomb-2019-0033 PMC7526024

[jnc70424-bib-0094] Souza Monteiro de Araujo, D. , R. Nassini , P. Geppetti , and F. de Logu . 2020. TRPA1 as a Therapeutic Target for Nociceptive Pain. Taylor & Francis. 10.1080/14728222.2020.1815191 PMC7610834 32838583

[jnc70424-bib-0095] Story, G. M. , A. M. Peier , A. J. Reeve , et al. 2003. “ANKTM1, a TRP‐Like Channel Expressed in Nociceptive Neurons, Is Activated by Cold Temperatures.” Cell 112: 819–829. 12654248 10.1016/s0092-8674(03)00158-2

[jnc70424-bib-0096] Sugiyama, D. , S. Kang , and T. J. Brennan . 2017. “Muscle Reactive Oxygen Species (ROS) Contribute to Post‐Incisional Guarding via the TRPA1 Receptor.” PLoS One 12: 1–17. 10.1371/journal.pone.0170410 PMC5245866 28103292

[jnc70424-bib-0097] Takizawa, M. , K. Harada , K. Nakamura , and T. Tsuboi . 2018. “Transient Receptor Potential Ankyrin 1 Channels Are Involved in Spontaneous Peptide Hormone Release From Astrocytes.” Biochemical and Biophysical Research Communications 501: 988–995. 29777700 10.1016/j.bbrc.2018.05.097

[jnc70424-bib-0098] Taylor, A. G. , T. G. Fischer‐White , J. G. Anderson , et al. 2016. “Stress, Inflammation and Pain: A Potential Role for Monocytes in Fibromyalgia‐Related Symptom Severity.” Stress and Health 32: 503–513. 27925450 10.1002/smi.2648

[jnc70424-bib-0099] Theoharides, T. C. , I. Tsilioni , and M. Bawazeer . 2019. “Mast Cells, Neuroinflammation and Pain in Fibromyalgia Syndrome.” Frontiers in Cellular Neuroscience 13: 1–8. 31427928 10.3389/fncel.2019.00353 PMC6687840

[jnc70424-bib-0100] Titiz, M. , L. Landini , D. Souza Monteiro de Araujo , et al. 2024. “Schwann Cell C5aR1 Co‐Opts Inflammasome NLRP1 to Sustain Pain in a Mouse Model of Endometriosis.” Nature Communications 15: 10142. 10.1038/s41467-024-54486-6 PMC11589863 39587068

[jnc70424-bib-0101] Trevisan, G. , S. Materazzi , C. Fusi , et al. 2013. “Novel Therapeutic Strategy to Prevent Chemotherapy‐Induced Persistent Sensory Neuropathy by TRPA1 Blockade.” Cancer Research 73: 3120–3131. 23477783 10.1158/0008-5472.CAN-12-4370

[jnc70424-bib-0102] Trevisani, M. , J. Siemens , S. Materazzi , et al. 2007. “4‐Hydroxynonenal, an Endogenous Aldehyde, Causes Pain and Neurogenic Inflammation Through Activation of the Irritant Receptor TRPA1.” Proceedings of the National Academy of Sciences 104: 13519–13524. 10.1073/pnas.0705923104 PMC1948902 17684094

[jnc70424-bib-0103] Tripathi, V. , A. Mishra , Y. Pathak , A. Jain , and H. Prakash . 2021. “Pathogenic Role of iNOs+ M1 Effector Macrophages in Fibromyalgia.” In Macrophages. IntechOpen.

[jnc70424-bib-0104] Üçeyler, N. , D. Zeller , A. K. Kahn , et al. 2013. “Small Fibre Pathology in Patients With Fibromyalgia Syndrome.” Brain 136: 1857–1867. 23474848 10.1093/brain/awt053

[jnc70424-bib-0105] Vandewauw, I. , K. de Clercq , M. Mulier , et al. 2018. “A TRP Channel Trio Mediates Acute Noxious Heat Sensing.” Nature 559: E7. 29539642 10.1038/nature26137

[jnc70424-bib-0106] Vittorio, S. , S. Erica , C. Cinzia , et al. 2020. “Comparison Between Acupuncture and Nutraceutical Treatment With Migratens in Patients With Fibromyalgia Syndrome: A Prospective Randomized Clinical Trial.” Nutrients 12: 821. 32204554 10.3390/nu12030821 PMC7146219

[jnc70424-bib-0107] Vriens, J. , and T. Voets . 2019. “Heat Sensing Involves a TR i Plet of Ion Channels.” British Journal of Pharmacology 176: 1–6. 10.1111/bph.14812 PMC6811738 31372975

[jnc70424-bib-0108] Wattiez, A. S. , L. P. Sowers , and A. F. Russo . 2020. “Calcitonin Gene‐Related Peptide (CGRP): Role in Migraine Pathophysiology and Therapeutic Targeting.” Expert Opinion on Therapeutic Targets 24: 91–100. 32003253 10.1080/14728222.2020.1724285 PMC7050542

[jnc70424-bib-0111] Wolfe, F. , D. J. Clauw , M. A. Fitzcharles , et al. 2010. “The American College of Rheumatology Preliminary Diagnostic Criteria for Fibromyalgia and Measurement of Symptom Severity.” Arthritis Care and Research 62: 600–610. 20461783 10.1002/acr.20140

[jnc70424-bib-0110] Wolfe, F. , D. J. Clauw , M. A. Fitzcharles , et al. 2011. “Fibromyalgia Criteria and Severity Scales for Clinical and Epidemiological Studies: A Modification of the ACR Preliminary Diagnostic Criteria for Fibromyalgia.” Journal of Rheumatology 38: 1113–1122. 21285161 10.3899/jrheum.100594

[jnc70424-bib-0109] Wolfe, F. , D. J. Clauw , M. A. Fitzcharles , et al. 2016. “2016 Revisions to the 2010/2011 Fibromyalgia Diagnostic Criteria.” Seminars in Arthritis and Rheumatism 46: 319–329. 27916278 10.1016/j.semarthrit.2016.08.012

[jnc70424-bib-0112] Wolfe, F. , H. A. Smythe , M. B. Yunus , et al. 1990. “The American College of Rheumatology 1990 Criteria for the Classification of Fibromyalgia.” Arthritis and Rheumatism 33: 160–172. 2306288 10.1002/art.1780330203

[jnc70424-bib-0113] Zhang, W. j. , S. c. Liu , L. g. Ming , et al. 2023. “Potential Role of Schwann Cells in Neuropathic Pain.” European Journal of Pharmacology 956: 175955. 37541365 10.1016/j.ejphar.2023.175955

